# The mediating role of circulating inflammatory cytokines in causal associations between plasma metabolites and asymptomatic bile duct and cholecyst calculus: A Mendelian randomization study

**DOI:** 10.1097/MD.0000000000041745

**Published:** 2025-03-07

**Authors:** Shayong Peng, Yongguang Wei, Huasheng Huang, Chenlu Lan, Zhiming Zeng, Guangzhi Zhu, Tao Peng

**Affiliations:** a Department of Hepatobiliary Surgery, The First Affiliated Hospital of Guangxi Medical University, Nanning, P.R. China; b Guangxi Key Laboratory of Enhanced Recovery after Surgery for Gastrointestinal Cancer, Nanning, P.R. China; c Key Laboratory of High-Incidence-Tumor Prevention and Treatment (Guangxi Medical University), Ministry of Education, Nanning, P.R. China.

**Keywords:** asymptomatic biliary tract calculus, asymptomatic gallstone, circulating inflammatory cytokines, genome-wide association study, Mendelian randomization, plasma metabolome

## Abstract

Asymptomatic gallbladder and biliary tract calculus may make into symptomatic disease or bring anxiety for patients. The formation of gallstones was associated with genetic risk factors and metabolic abnormalities. Genome-wide association studies (GWAS) data of 1400 plasma metabolites (PMs) and 91 circulating inflammatory cytokines (CICs) were obtained from the GWAS catalog, while the GWAS data of calculus of gallbladder without cholecystitis and calculus of bile duct without cholangitis or cholecystitis were retrieved from the IEU OpenGWAS project. The causalities from PMs or CICs to asymptomatic bile duct or cholecyst calculus were explored by 2-sample Mendelian randomization (MR) analysis. Furthermore, the MR analyses were implemented from the identified PMs to CICs. Following the false discovery rate adjustment, the significant causalities, including 6 CICs and 5 PMs on asymptomatic biliary stone and 5 CICs and 48 PMs on asymptomatic gallstone, were identified. Fibroblast growth factor 19 (FGF-19) and aspartate/mannose ratio were the common protective factors of asymptomatic biliary tract calculus, while Monocyte chemoattractant protein 2 (CCL-2) may serve as a disease-promoting agent. Moreover, Bilirubin degradation product, C17H18N2O4 (1) levels, and Bilirubin (Z,Z)/etiocholanolone glucuronide ratio were associated with FGF-19 level, while aspartate/mannose ratio was related to TNF-related apoptosis-inducing ligand level. Based on MR analysis, we identified the multiple PMs and CICs, especially FGF-19, which may affect the formation of gallbladder and biliary tract calculus. Moreover, the partial CICs could be the downstream mediator of PMs related to asymptomatic gallbladder and biliary tract calculus. These results contributed to supporting previous studies and provided evidence for disease prevention or management.

## 1. Introduction

Gallstones in the gallbladder or biliary tract are common in adulthood, and they occur in approximately 10% to 20% of the global adult population.^[1,2]^ The prevalence of gallstone disease is increasing, largely due to the global epidemic of obesity associated with insulin resistance, a key feature of the metabolic syndrome.^[1,3]^ Most stones can be asymptomatic, while > 20% of people with gallstones will develop symptoms in their lifetime.^[1,3]^ Conventional management of asymptomatic gallbladder and biliary tract calculus is mainly the recommendation of a “watch and wait” approach, including regular checkups and monitoring for any changes or development of symptoms over time.^[4]^ Although surgery is generally not advised for gallstones that do not cause any symptoms, it may be taken into consideration in specific circumstances, such as when complications are likely to occur or when the patient has other underlying medical issues that could raise the likelihood of future symptoms.^[4]^ Asymptomatic gallbladder and biliary tract calculus may make into symptomatic disease or bring anxiety for patients. Therefore, exploring the genesis of gallstones makes sense in the prevention or management of this underlying disease.

According to the composition and appearance, gallstones are categorized as cholesterol gallstones (the predominant 1), pigment gallstones, and mixed gallstones, in which the brown pigment stones tend to form in the unsmooth biliary tract with infection, while the black is associated with hemolytic anemia. The crucial factor of cholesterol gallstone remains believed to be the phase separation of cholesterol crystals from supersaturated bile. As for pigment gallstones, the supersaturation of bile with calcium hydrogen bilirubinate plays an important factor. Imbalance between the cholesterol, bile salts, and phospholipids, that is, hypersecretion of hepatic cholesterol into bile accompanied by less frequent hyposecretion of bile salts and/or phospholipids, is the primary pathophysiological defect of cholesterol gallstones.^[5–8]^ A cross-sectional analysis demonstrated that serum cotinine levels and tobacco exposure were the contributing agents for gallstones.^[9]^ It is demonstrated that homocysteine levels are often elevated in patients with metabolic syndrome, and this abnormality could lead to a proinflammatory condition, which contributes to the formation of biliary stones.^[10]^ The presence of neutrophil extracellular traps was found in human and murine gallstones.^[11]^ In addition, gene mutations of adenosine triphosphate (ATP)-binding cassette (ABC) subfamily B member 4 (ABCB4) make sense in the formation of cholesterol gallstones.^[12,13]^ During passage through the digestive tract, enterohepatic circulation effectively recycles.^[2]^ Intestinal disorders, such as Crohn Disease, may result in high efficiency of cholesterol absorption or facilitate the conversion from primary bile acids (BAs) to secondary BAs.^[14,15]^ By 16S rRNA gene sequencing, Wang et al found that intestinal flora imbalance affects bile acid and cholesterol metabolism and is associated with gallstone formation.^[16]^ Lithogenic diet led to the formation of cholesterol gallstones in mice, and the pathogenesis may be the gut microbiota dysbiosis,^[17,18]^ such as the decreased Firmicutes and ratio of Firmicutes to Bacteroidetes.^[18]^ Nevertheless, the changes in levels of body metabolites and inflammatory cytokines in patients with gallstones need to be further elaborated.

Mendelian randomization (MR) analysis is a genetic approach and was widely employed in uncovering the causal estimates between exposure and outcome. The previous study had tried to reveal the contributing role of serum polyunsaturated fatty acids for the occurrence of calculus of the gallbladder or cholecystitis.^[19]^ Utilizing the MR approach, we managed to seek the potential circulating metabolites and inflammatory cytokines associated with asymptomatic gallstone, which may aid in the development of prevention strategies and therapeutic modalities.

## 2. Materials and methods

### 2.1. Genome-wide association study data of 1400 plasma metabolites and 91 circulating inflammatory cytokines

As the exposure data in MR analysis, the genome-wide association study (GWAS) data sets of 1400 plasma metabolites (PMs), including 1091 blood metabolites and 309 metabolite ratios, were retrieved from a GWAS summary study, which originated from 8299 individuals involved in the Canadian longitudinal study of aging (CLSA) cohort study.^[20]^ The CLSA summary statistics is an exhaustive study on compounds from the serum metabolome linked to human disorders, and it can be downloaded in the GWAS Catalog (accession numbers from GCST90274758 to GCST90274848; https://www.ebi.ac.uk/gwas/). The serum metabolites were ascribed into 8 major metabolic categories, containing lipid, amino acid, and peptide and so on.

In addition, the summary statistics data for 91 circulating inflammatory cytokines (CICs) can also be obtained in the GWAS Catalog (accession numbers: GCST90274758 to GCST90274848; https://www.ebi.ac.uk/gwas/), and the genetic instruments of CICs originate from a genome-wide protein quantitative trait locus study. This comprehensive study included 14,824 European-ancestry participants and employed the Olink Target Inflammation Panel in protein measurements.^[21]^

### 2.2. Genome-wide association study data of calculus of gallbladder without cholecystitis and calculus of bile duct without cholangitis or cholecystitis

As the outcome data of MR analysis, summary GWAS data for calculus of bile duct without cholangitis or cholecystitis were extracted from the IEU Open GWAS project (GWAS ID: ukb-b-8268, Trait name: Calculus of bile duct without cholangitis or cholecystitis; Published by Ben Elsworth et al, https://gwas.mrcieu.ac.uk/). This summary statistic contains 1706 cases and 461,304 controls of male or female Europeans, and the total number of single-nucleotide polymorphisms (SNPs) is 9851,867.

Additionally, we further retained GWAS data of calculus of gallbladder without cholecystitis from the IEU Open GWAS project (GWAS ID: ukb-b-11020, Trait name: Calculus of bile duct without cholangitis or cholecystitis; published by Ben Elsworth et al, https://gwas.mrcieu.ac.uk/). The genetic instruments of calculus of gallbladder without cholecystitis come from the 5766 European cases and 457,244 European controls.

### 2.3. Study design

**Figure 1.**
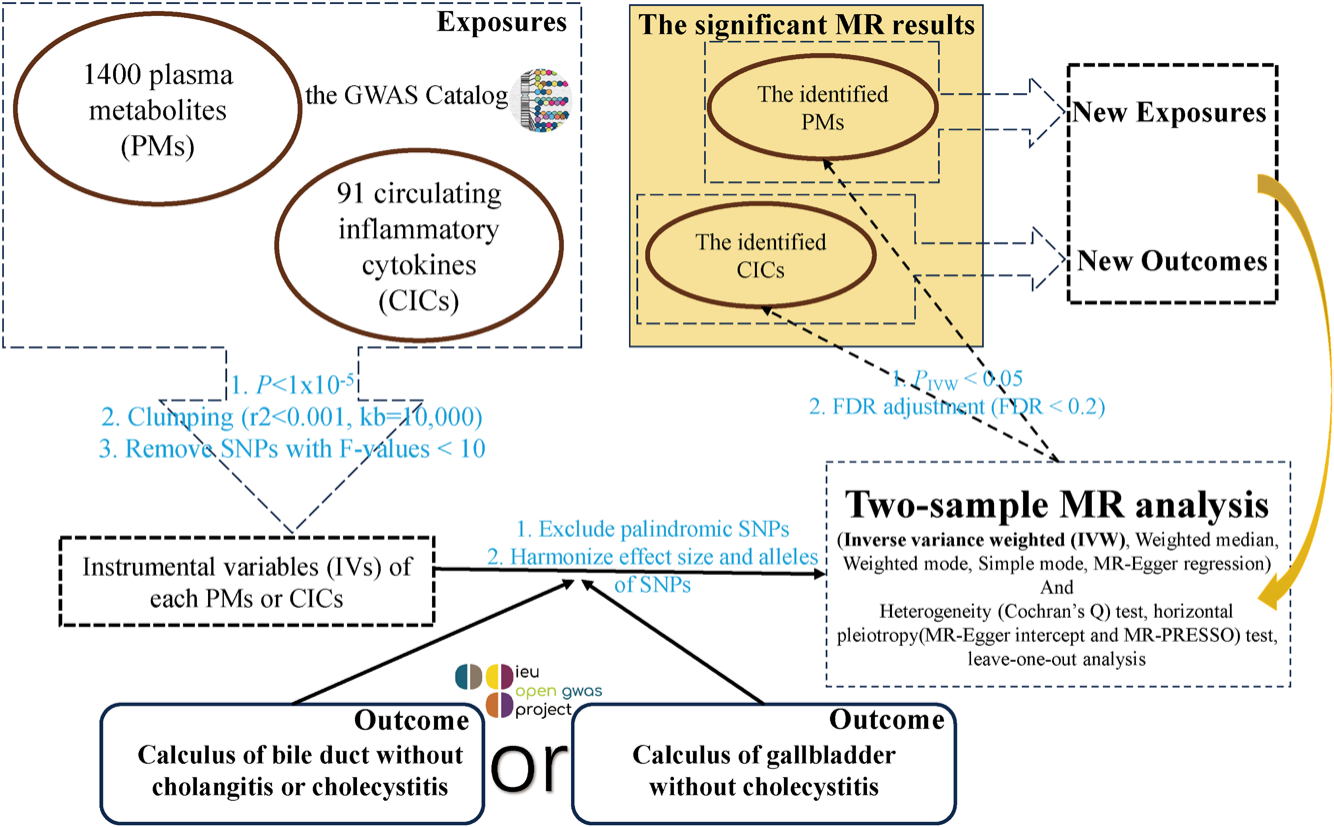
The overall design of study. GWAS = genome-wide association study, MR = Mendelian randomization, MR-PRESSO = Mendelian randomization pleiotropy residual sum and outlier, SNPs = single-nucleotide polymorphisms.

Firstly, to explore the related metabolic substance and inflammatory mediators of asymptomatic gallbladder and biliary tract calculus, the 2-sample MR analyses were carried out from exposures (1400 PMs and 91 CICs) to outcomes (calculus of gallbladder without cholecystitis or calculus of bile duct without cholangitis or cholecystitis). Subsequently, the causal associations from the identified PMs related to asymptomatic gallstone or biliary stone to the identified CICs were probed into using the 2-sample MR analysis. The overall design of the study was shown in Figure 1. Strengthening the reporting of observational studies in epidemiology using MR principles were followed in our study.^[22]^ The following basic prerequisites need to be met by the SNPs assumed to represent the IVs for MR analysis. There must be a substantial correlation between the SNPs and the exposure issue. SNPs and outcomes should not be related through confounding factors. The result should not be impacted by the SNPs directly.

### 2.4. Instrumental variables screening for GWAS data of exposure

Regarding the screening of instrumental variables (IVs) for exposure, we adopted a more permissive cutoff, a *P*-value of 1 × 10^−5^, to identify the SNPs correlated with every PM and CIC for further MR analysis, potentially improving the viability.^[23–25]^ Subsequently, employing linkage disequilibrium (LD) analysis (parameters: LD r² < 0.001 and distance > 10,000 kb), the screened genetic instruments for each PM or CIC were clumped and determined additionally, in which the 1000 Genomes Project European samples were taken as the LD reference panel.^[26]^ Besides, the SNPs not present in the panel were also eliminated.^[27]^ These processes contributed to preventing bias in the causalities. In MR analysis, the F-statistic is utilized to evaluate the validity of the IVs by assessing their strength and potential for bias in causal inference.^[28]^ The F-statistics for SNPs in each PM and CIC were figured out. Then, we included the IVs with the F-statistic < 10 for further 2-sample MR analysis.

### 2.5. Mendelian randomization estimates and reverse MR analysis

Subsequently, the effect variants, that is, the eventually screened IVs, of PMs or CICs were harmonized with the SNPs information in GWAS data of outcome (calculus of bile duct without cholangitis or cholecystitis, calculus of gallbladder without cholecystitis or the identified CICs), wherein the SNPs being palindromic with intermediate allele frequencies were eliminated. Then, the 2-sample MR analysis was further performed based on the harmonized data, and only the exposure (each PM or CIC) with > three IVs can be included. The statistical methods of our MR analysis contained inverse variance weighted (IVW), Weighted median, Weighted mode, Simple mode, and MR-Egger regression approaches, in which IVW method acted as the dominate 1.^[29]^ And the MR-Egger regression contributed to examining the reliability of IVW results. As another supplementary MR method, the weighted median method required that the number of IVs of an exposure should be up to half of candidate SNPs. Therefore, the statistically significant causality was determined if the *P*_IVW_ < 0.05. Besides, the false discovery rate (FDR) of the estimated causality of MR analysis was further calculated to control the proportion of false positive results.^[30]^ The causal effect was further considered to be significant if its FDR < 0.2, and OR values of various MR approaches were basically all > 1 or < 1. In addition, weighted mode and simple mode methods were further performed to promote consistency and preciseness. Our comprehensive MR analyses were performed in the R program (V4.3.1), in which “TwoSampleMR,” “VariantAnnotation,” and “gwasglue” served as the main R packages.

### 2.6. Sensitivity analyses

For the estimated causal assumptions, the existence of heterogeneity between genetic instruments will erode their power. Hence, Cochran *Q* tests for the IVW and MR-Egger results were carried out. Additionally, horizontal pleiotropy was also detected by calculating the MR-Egger intercept and its corresponding *P*-value. We can assume that there was the presence of significant heterogeneity or horizontal pleiotropy in the event that the *P*-value of Cochran *Q* test or MR-Egger intercept > .05. Moreover, the horizontal pleiotropy was further examined and modified by MR Pleiotropy Residual Sum and Outlier (MR-PRESSO) global tests, in which the SNP outliers were identified and removed.^[31]^ Eventually, to guarantee the robustness, the “leave-one-out” analysis was implemented to detect the influence of single IVs on each casual estimates.

## 3. Results

In this study, approximately 15.4 million SNPs were contained in the GWAS summary statistics for 1400 PMs. Then, by multiple screening steps, a total of 34,843 SNPs for the 1091 blood metabolites and 309 metabolite ratios were involved in the 2-sample MR analysis. The detailed PMs information was shown (Table S1, Supplemental Digital Content, http://links.lww.com/MD/O459). In addition, a total of 1815 SNPs for 91 CICs were retained after screening, and the summary for these CICs was presented in Table S2, Supplemental Digital Content, http://links.lww.com/MD/O459.

### 3.1. The causal effects of CICs on asymptomatic gallbladder and biliary tract calculus

**Figure 2.**
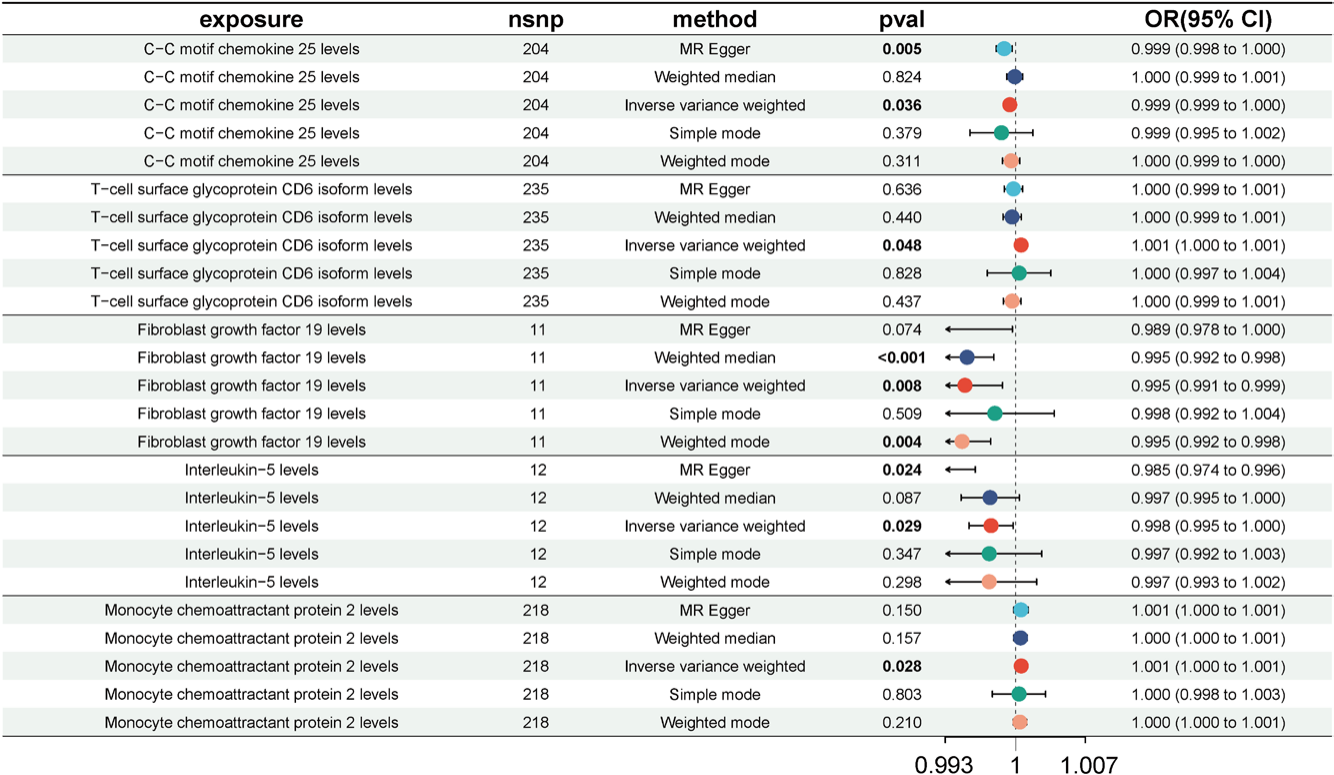
Forest plots illustrating the significant casual estimates (*P*_IVW_ < .05 and FDR < 0.2) of 2-sample MR analyses from CICs on asymptomatic biliary tract calculus. 95% CI = 95% confidence interval, CICs = circulating inflammatory cytokines, IVW = inverse variance weighted, MR = Mendelian randomization, OR = odd ratio.

After FDR adjustment, we found that the genetically predicted C-C motif chemokine 25 (CCL-25; OR = 0.9994, 95% CI = 0.99885–0.99996, *P*_IVW_ = .036), Fibroblast growth factor 19 (FGF-19; OR = 0.9949, 95% CI = 0.9912–0.9987, *P*_IVW_ = .008), and interleukin-5 (IL-5; OR = 0.9975, 95% CI = 0.9953–0.9997, *P*_IVW_ = .029) levels were the protective factor of asymptomatic gallbladder calculus, while T-cell surface glycoprotein CD6 isoform (CD6; OR = 1.0006, 95% CI = 1.000–1.001, *P*_IVW_ = .048) and monocyte chemoattractant protein 2 (CCL-2; OR = 1.0006, 95% CI = 1.000–1.001, *P*_IVW_ = .028) levels indicated a contributing role of asymptomatic biliary tract calculus risk (Fig. 2 and Table S3, Supplemental Digital Content, http://links.lww.com/MD/O459). The corresponding scatter plots of these 5 casual effects were illustrated in Figure S1A–E, Supplemental Digital Content, http://links.lww.com/MD/O458.

**Figure 3.**
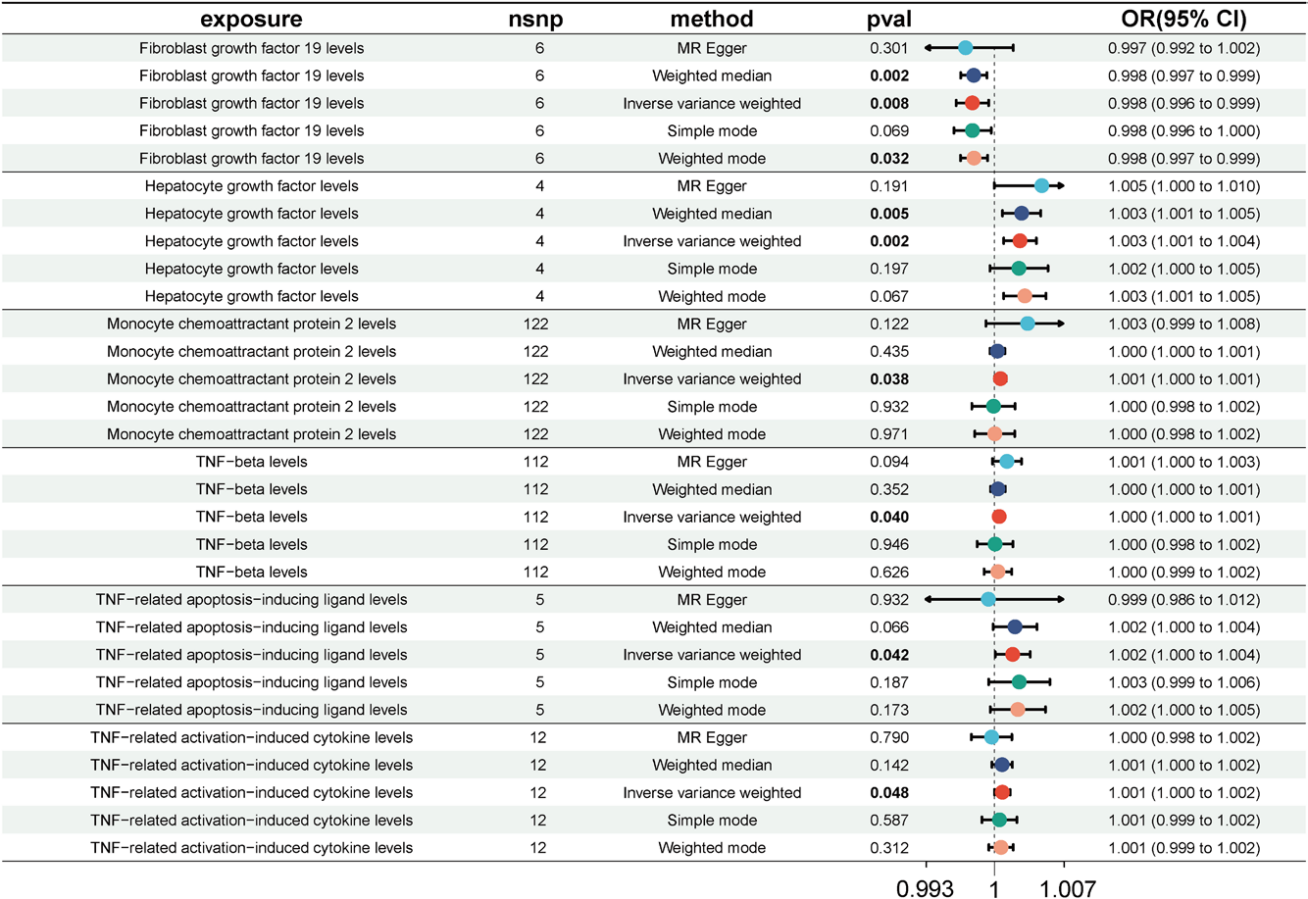
Forest plots illustrating the significant casual estimates (*P*_IVW_ < .05 and FDR < 0.2) of 2-sample MR analyses from CICs on asymptomatic gallbladder calculus. 95% CI = 95% confidence interval, CICs = circulating inflammatory cytokines, IVW = inverse variance weighted, MR = Mendelian randomization, OR = odd ratio.

Besides, consistent with CICs associated with asymptomatic gallbladder calculus, the genetically predicted FGF-19 (OR = 0.998, 95% CI = 0.996–0.999, *P*_IVW_ = .008) was also the protective factor of asymptomatic biliary tract calculus, while Monocyte chemoattractant protein 2 (CCL-2; OR = 1.0006, 95% CI - 1.000–1.001, *P*_IVW_ = .038) may serve as a disease-promoting agent (Fig. 3 and Table S4, Supplemental Digital Content, http://links.lww.com/MD/O459). The other 4 CICs, including hepatocyte growth factor (HGF; OR = 1.003, 95% CI = 1.001–1.004, *P*_IVW_ = .002), TNF-beta (TNFB; OR = 1.0005, 95% CI = 1.000–1.001, *P*_IVW_ = .040), TNF-related apoptosis-inducing ligand (TRAIL; OR = 1.0019, 95% CI = 1.000–1.0037, *P*_IVW_ = .042), and TNF-related activation-induced cytokine (TRANCE; OR = 1.0008, 95% CI = 1.000–1.0016, *P*_IVW_ = .048) levels, were favorable for an increased risk of asymptomatic gallbladder calculus (Fig. 3 and Table S4, Supplemental Digital Content, http://links.lww.com/MD/O459). The corresponding scatter plots of these 5 casual effects were illustrated in Figure S2A to F, Supplemental Digital Content, http://links.lww.com/MD/O458.

### 3.2. The causal effects of PMs on asymptomatic gallbladder and biliary tract calculus

**Figure 4.**
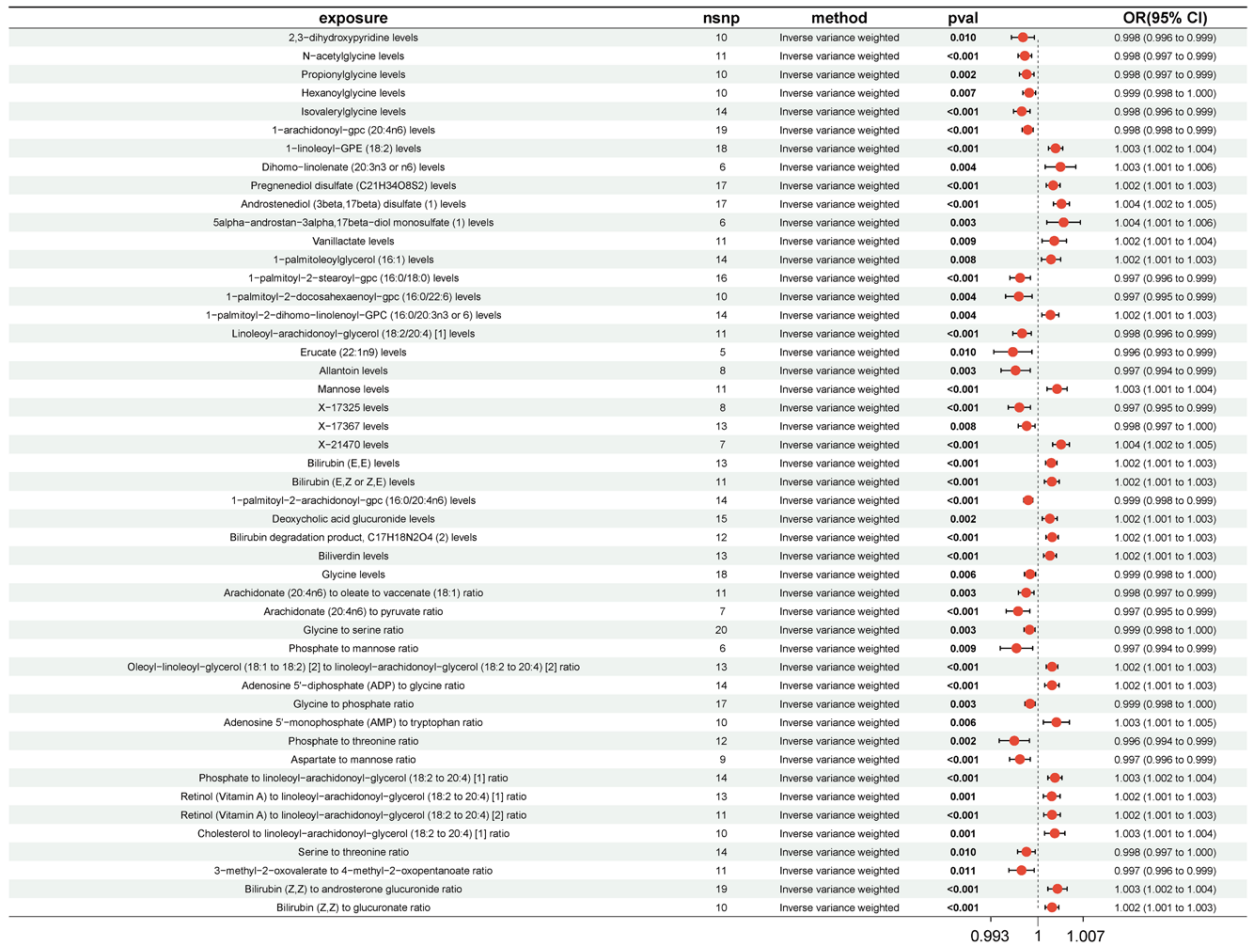
Forest plots illustrating the significant casual estimates (*P*_IVW_ < .05 and FDR < 0.2) of 2-sample MR analyses from PMs on asymptomatic biliary tract calculus. 95% CI = 95% confidence interval, IVW = inverse variance weighted, MR = Mendelian randomization, OR = odd ratio, PMs = plasma metabolites.

Following the FDR adjustment, the IVW results showed that the 5 PMs had the casual associations with asymptomatic biliary stone, in which bilirubin degradation product, C17H18N2O4 (1 and 3) levels (OR = 1.001, 95% CI = 1.000–1.001, *P*_IVW_ < .01; OR = 1.001, 95% CI = 1.000–1.001, *P*_IVW_ = .01), bilirubin (z,z) levels (OR = 1.001, 95% CI = 1.000–1.001, *P*_IVW_ < .01) and bilirubin (Z,Z)/etiocholanolone glucuronide ratio (OR = 1.001, 95% CI = 1.000–1.002, *P*_IVW_ = .01) indicated a contributing role on asymptomatic biliary stone risk, while aspartate/mannose ratio (OR = 0.998, 95% CI = 0.998–0.999, *P*_IVW_ < .01) showed a protective impact. Furthermore, these significant associations remained stable in weighted median and weighted mode methods (Fig. 4 and Table S5, Supplemental Digital Content, http://links.lww.com/MD/O459). The corresponding scatter plots of these 5 casual effects were illustrated in Figure S3A to E, Supplemental Digital Content, http://links.lww.com/MD/O458.

**Figure 5.**
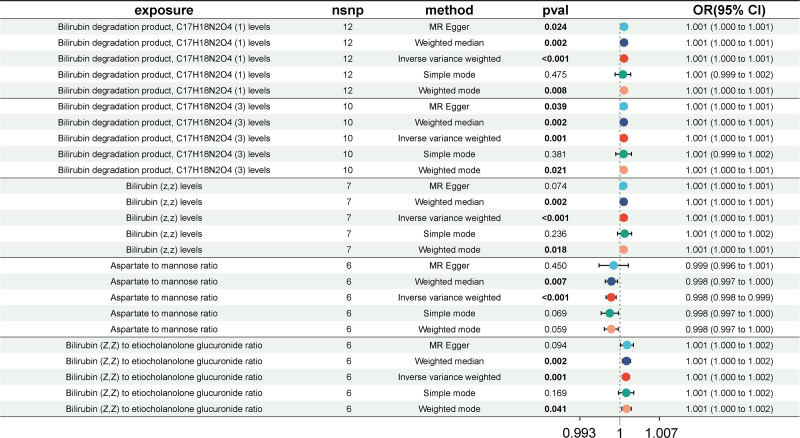
Forest plots illustrating the significant casual estimates (*P*_IVW_ < .05 and FDR < 0.2) of 2-sample MR analyses from the PMs on asymptomatic gallbladder calculus. 95% CI = 95% confidence interval, IVW = inverse variance weighted; MR, Mendelian randomization; OR, odd ratio, PMs = plasma metabolites.

Additionally, in terms of asymptomatic gallbladder stone, a total of 48 PMs were identified, and these casual estimates were also supported by other MR methods (Fig. 5 and Table S6, Supplemental Digital Content, http://links.lww.com/MD/O459). The according scatterplots were presented in Figure S4, Supplemental Digital Content, http://links.lww.com/MD/O458. Of the 48 PMs identified, the genetically predicted Erucate (22:1n9) levels were found to have the smallest OR value (OR = 0.9962, 95% CI = 0.9932–0.9991, *P*_IVW_ = .01), which suggested that it may carry the strongest preventative impact, while 5alpha-androstan-3alpha, 17beta-diol monosulfate (1) levels had the strongest contributing effect on calculus of gallbladder without cholecystitis (OR = 1.0039, 95% CI = 1.0013–1.0065, *P*_IVW_ = .0013). Noticeably, aspartate/mannose ratio was the common and protective factor of asymptomatic gallbladder and biliary stone risk.

### 3.3. Revealing the potential causalities from the identified PMs to CICs

Body metabolism and substance synthesis can affect the inflammatory mediators in local organs or throughout the body.^[32]^ To identify downstream CICs of PMs for the asymptomatic gallbladder and biliary tract calculus, the 2-sample MR analysis was performed from the aforementioned PMs casually related to the asymptomatic gallbladder and biliary tract calculus to the identified CICs. The results of the IVW method revealed that the genetically predicted Bilirubin degradation product, C17H18N2O4 (1) levels, and Bilirubin (Z,Z)/etiocholanolone glucuronide ratio were associated with FGF-19 level. And aspartate/mannose ratio were related to TRAIL level (Table S7, Supplemental Digital Content, http://links.lww.com/MD/O459). As for asymptomatic biliary stone, the multiple causalities from the identified PMs to CICs were uncovered, and the detailed casual estimates were shown in Table S8, Supplemental Digital Content, http://links.lww.com/MD/O459.

### 3.4. Sensitivity and pleiotropy analysis

To further examination, the robustness of the above-mentioned causalities, the sensitivity analysis was conducted to detected the heterogeneity and horizontal pleiotropy of the identified IVs. Heterogeneity test results of IVW and MR-Egger methods demonstrated that no heterogeneity were detected, except the CCL25 levels, Bilirubin (E,Z or Z,E) levels, 5alpha-androstan-3alpha,17beta-diol monosulfate (1) levels, and bilirubin (Z,Z) to androsterone glucuronide ratio for calculus of gallbladder without cholecystitis, and the FGF-19 levels for calculus of bile duct without cholangitis or cholecystitis [Fig. S5–8, Supplemental Digital Content (http://links.lww.com/MD/O458) and Table S3–8, Supplemental Digital Content (http://links.lww.com/MD/O459)]. The MR-Egger intercept tests showed almost no evidence of horizontal pleiotropy, except in the analyses of CD6 and IL-5 levels for calculus of gallbladder without cholecystitis. However, except for the CCL25 levels, FGF-19 levels, Bilirubin (Z,Z)/androsterone glucuronide ratio, Phosphate/threonine ratio, and 5alpha-androstan-3alpha,17beta-diol monosulfate (1) levels for calculus of gallbladder without cholecystitis, MR-PRESSO global tests did not reveal any significant outliers (*P* > .05, Table S6, Supplemental Digital Content, http://links.lww.com/MD/O459). In addition, when the outlier SNPs were excluded, outlier-corrected MR-PRESSO analysis did not disclose any horizontal pleiotropy of these IVs (Table S6, Supplemental Digital Content, http://links.lww.com/MD/O459). The MR leave-one-out sensitivity analysis indicated that sequentially excluding individual SNP did not significantly influence the results, in which all the estimates of the error lines were on the same side, except for a SNP (rs174564) of Arachidonate (20:4n6) to oleate to vaccenate (18:1) ratio (Fig. S9–12, Supplemental Digital Content, http://links.lww.com/MD/O458).

## 4. Discussion

An imbalance in the composition of bile is the crucial factor in the calculus of the gallbladder, and biliary tract, and the alterations of circulating metabolites and inflammatory cytokines may influence the bile composition. For serum lipids, serum low-density lipoprotein cholesterol and high-density lipoprotein cholesterol levels could be inversely related to cholelithiasis risk, and lower serum total cholesterol can causally increase the risk.^[8]^ Gallstone development is considerably more prevalent in women than in men, which suggests that some female sex hormones may facilitate the formation of gallstone.^[7]^ Wang et al demonstrated that Glutaredoxin-1 (Glrx1) and Glrx1-modulated protein S-glutathionylation increased the risk of gallstone occurrence, which may result from ASGR1-LXRα-dependent extrusion and bile-acid-dependent resorption of cholesterol.^[33]^ In addition, gallstone genesis may be influenced by phthalate metabolites through modifications to the secretion of insulin.^[34]^ Using the MR approach, Hu et al found that Omega-3 polyunsaturated fatty acids may serve as a mediating role in the contributing effect of the Genus Peptococcus on gallstone disease.^[35]^ In our study, FGF-19 exhibited the protective impacts in both asymptomatic biliary tract and gallbladder calculus, while CCL2 indicated a contributing role. It is well known that FGF-19, an intestinal hormone, can sense the ileal reabsorption of BAs to control the hepatic synthesis of Bas.^[36,37]^ Normally, FGF-19 helps to suppress BAs synthesis in the liver by inhibiting the enzyme cholesterol 7 alpha-hydroxylase (CYP7A1), while such a regulatory mechanism may not function properly, leading to an imbalance in BAs production and potentially contributing to the formation of gallstones in individuals with gallstone disease.^[38]^ And rs6471717 near CYP7A1 was demonstrated to be important SNPs of gallstone disease risk.^[39]^ Nevertheless, a study demonstrated that, in overweight individuals without gallstone disease, the regulation of bile acid synthesis via FGF-19 typically remains intact, allowing for proper metabolic control and reducing the risk of gallstone formation.^[36]^ This difference highlights the potential importance of FGF-19 in maintaining bile acid homeostasis and its impact on gallstone disease pathology.

Lilly Kristin Kunzmann et al illustrated that, for primary sclerosing cholangitis patients, microbe-activated monocytes induced the secretion of Th17 and monocyte-recruiting chemokines chemokine (C-C motif) ligand (CCL)-20 and CCL-2 in human primary cholangiocytes.^[40]^ And there is no recognized role of CCL25 and IL-5 in the pathophysiology of gallstone disease. In addition, our study indicated that some TNF-related CICs were the accelerative factors of asymptomatic gallstone. It is demonstrated that the increased expression of these genes and TNF-a was believed to be associated with gallstone disease.^[41,42]^ Calcium bilirubinate salts, the combination of biliary bilirubin and calcium, are the predominant constituent of pigment gallstones. Our study also demonstrated that multiple circulating bilirubin-related metabolites carried the causalities with asymptomatic biliary tract and gallbladder calculus risk. Actually, the previous studies had found this casual association.^[43–46]^ Noticeably, most plasm metabolites associated with bile duct stone were bilirubin-related metabolites, including bilirubin levels, bilirubin degradation products, and bilirubin/etiocholanolone glucuronide ratio. The revelation of circulating metabolites and inflammatory mediators was conducive to developing the prevention strategies and therapeutic modalities for gallstone individuals. Making dietary changes, such as reducing the intake of fatty foods, can sometimes help prevent the progression of asymptomatic gallstones into symptomatic ones.^[47–49]^ In a drug-target MR analysis, HMGCR inhibition contributed to reducing the cholelithiasis risk.^[8]^ Moreover, through the activation of PPARα-mediated CYP7A1 expression and the facilitation of the conversion of cholesterol into BAs, PCSK9 inhibition has both preventative and therapeutic effects on cholesterol gallstones.^[50]^ Our study has some potential limitations. Firstly, a relatively loose screening *P*-value was adopted, which may lead to the SNPs of exposures that were not so relevant being included. Secondly, the 4 summary GWAS data were all based on an individual of European ancestry. Therefore, whether the conclusion is applicable in other ethnicities still needs further analysis and verification.

## 5. Conclusions

In conclusion, our study reinforces the possible causative involvement of circulating metabolites and inflammatory mediators in the formation of asymptomatic gallbladder and biliary tract calculus. FGF-19 and aspartate/mannose ratio were the common protective factors of asymptomatic biliary tract calculus, while CCL2 may serve as a disease-promoting agent. These insights enhance our knowledge of gallstones and aid in developing prevention strategies and therapeutic modalities.

## Acknowledgments

Firstly, I would like to express my gratitude to the authors of this article and thank them for their help and dedication from the end. Second, we would like to thank the contributors of the public database included in our study. Finally, the authors would like to thank all the staff in the editorial department for all your valuable comments and responsible work.

## Author contributions

**Conceptualization:** Shayong Peng, Tao Peng.

**Formal analysis:** Shayong Peng, Yongguang Wei, Chenlu Lan.

**Funding acquisition:** Guangzhi Zhu, Tao Peng.

**Investigation:** Yongguang Wei, Zhiming Zeng.

**Methodology:** Shayong Peng, Yongguang Wei, Chenlu Lan.

**Project administration:** Guangzhi Zhu, Tao Peng.

**Resources:** Yongguang Wei, Huasheng Huang, Chenlu Lan.

**Software:** Yongguang Wei, Huasheng Huang, Chenlu Lan.

**Supervision:** Zhiming Zeng, Guangzhi Zhu, Tao Peng.

**Validation:** Shayong Peng, Huasheng Huang.

**Visualization:** Huasheng Huang.

**Writing – original draft:** Shayong Peng.

**Writing – review & editing:** Shayong Peng, Huasheng Huang, Zhiming Zeng, Guangzhi Zhu, Tao Peng.

## Supplementary Material
