# Supplementary material for: The mediating role of circulating inflammatory cytokines in causal associations between plasma metabolites and asymptomatic bile duct and cholecyst calculus: A Mendelian randomization study
Source: Medicine (Baltimore). 2025 Mar 7;104(10):e41745. doi: 10.1097/MD.0000000000041745 (PMC11902928; doi:10.1097/MD.0000000000041745)
Supplement: Supplementary file 2 [file medi-104-e41745-s002.pdf]

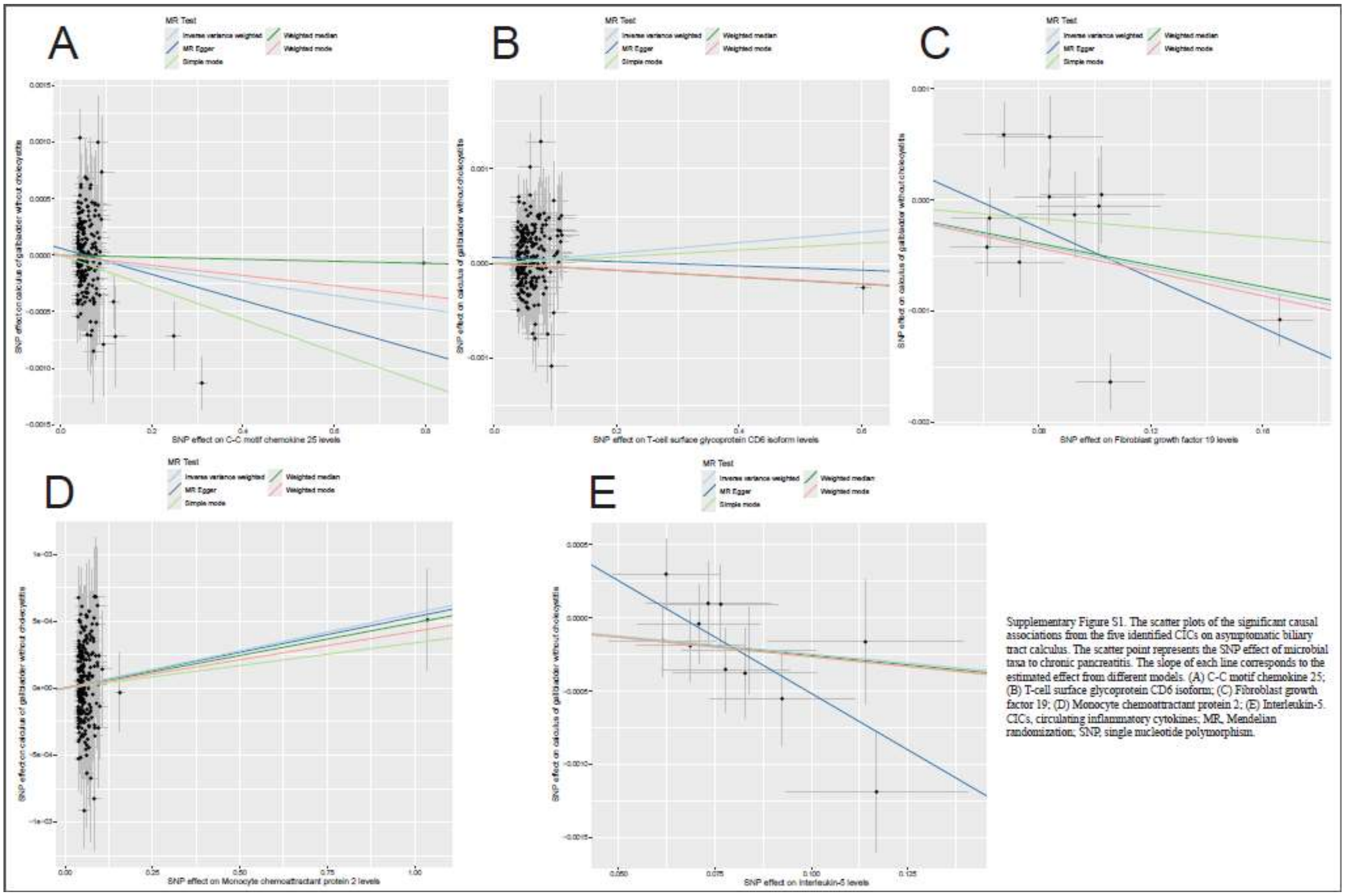

Supplementary Figure S1. The scatter plots of the significant causal associations from the five identified CICs on asymptomatic biliary tract calculus. The scatter point represents the SNP effect of microbial taxa to chronic pancreatitis. The slope of each line corresponds to the estimated effect from different models. (A) C-C motif chemokine 25; (B) T-cell surface glycoprotein CD6 isoform; (C) Fibroblast growth factor 19; (D) Monocyte chemoattractant protein 2; (E) Interleukin-5. CICs, circulating inflammatory cytokines; MR, Mendelian randomization; SNP, single nucleotide polymorphism.

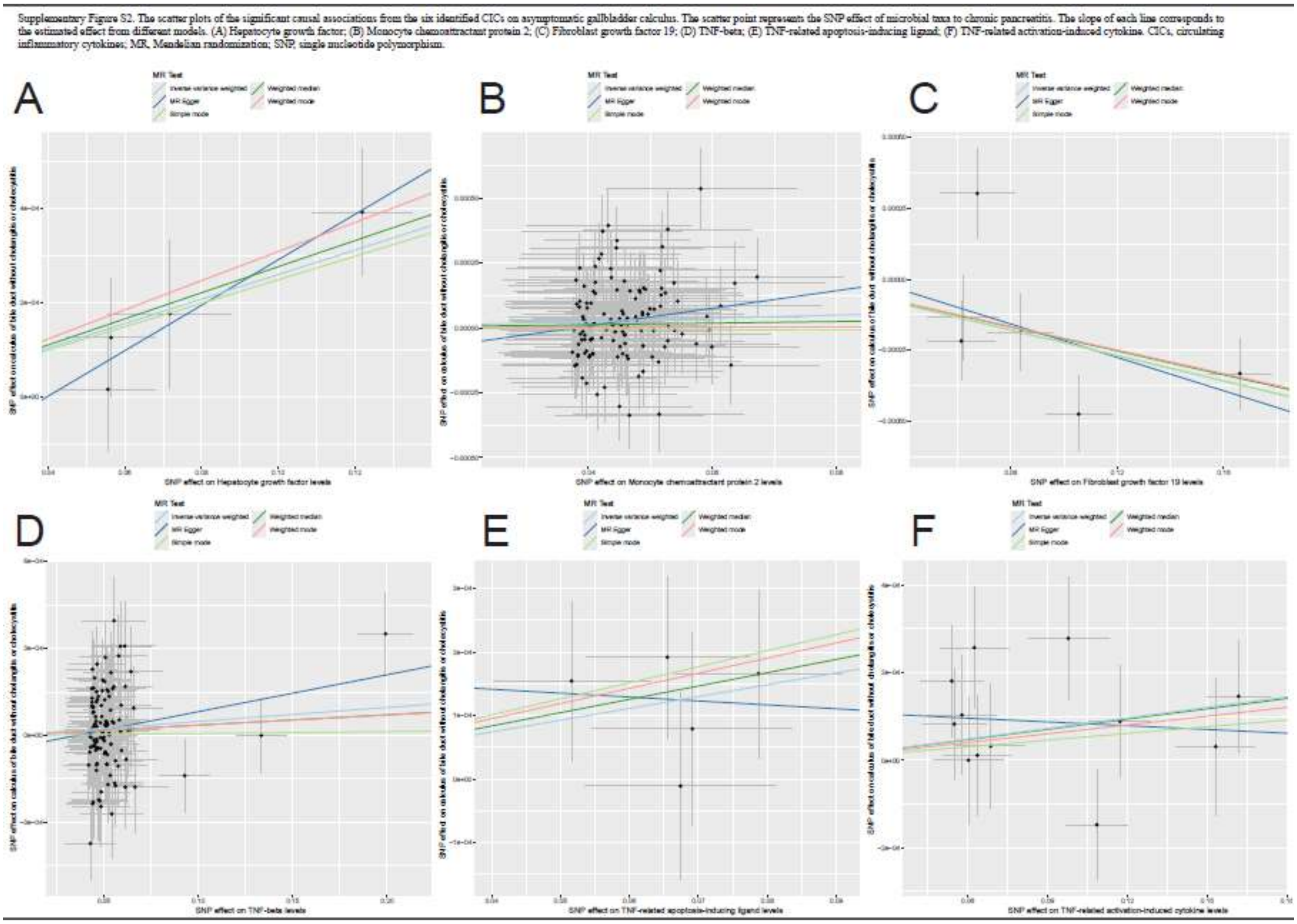

Supplementary Figure S2. The scatter plots of the significant causal associations from the six identified CICs on asymptomatic gallbladder calculus. The scatter point represents the SNP effect of microbial taxa to chronic pancreatitis. The slope of each line corresponds to the estimated effect from different models. (A) Hepatocyte growth factor; (B) Monocyte chemoattractant protein 2; (C) Fibroblast growth factor 19; (D) TNF-beta; (E) TNF-related apoptosis-inducing ligand; (F) TNF-related activation-induced cytokine. CICs, circulating inflammatory cytokines; MR, Mendelian randomization; SNP, single nucleotide polymorphism.

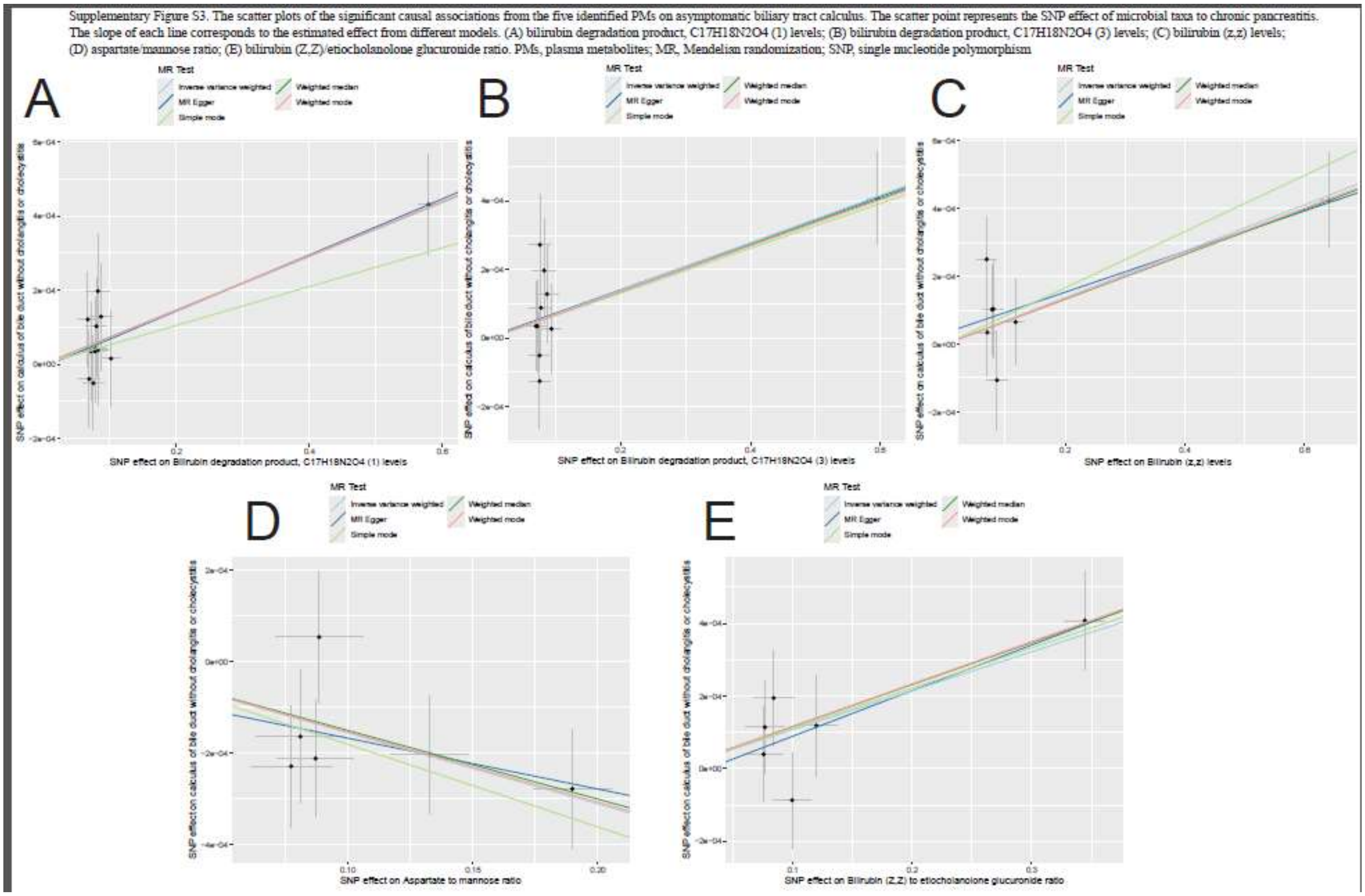

Supplementary Figure S3. The scatter plots of the significant causal associations from the five identified PMs on asymptomatic biliary tract calculus. The scatter point represents the SNP effect of microbial taxa to chronic pancreatitis. The slope of each line corresponds to the estimated effect from different models. (A) bilirubin degradation product, C17H18N2O4 (1) levels; (B) bilirubin degradation product, C17H18N2O4 (3) levels; (C) bilirubin (z,z) levels; (D) aspartate/mannose ratio; (E) bilirubin (Z,Z)/etiocholanolone glucuronide ratio. PMs, plasma metabolites; MR, Mendelian randomization; SNP, single nucleotide polymorphism.

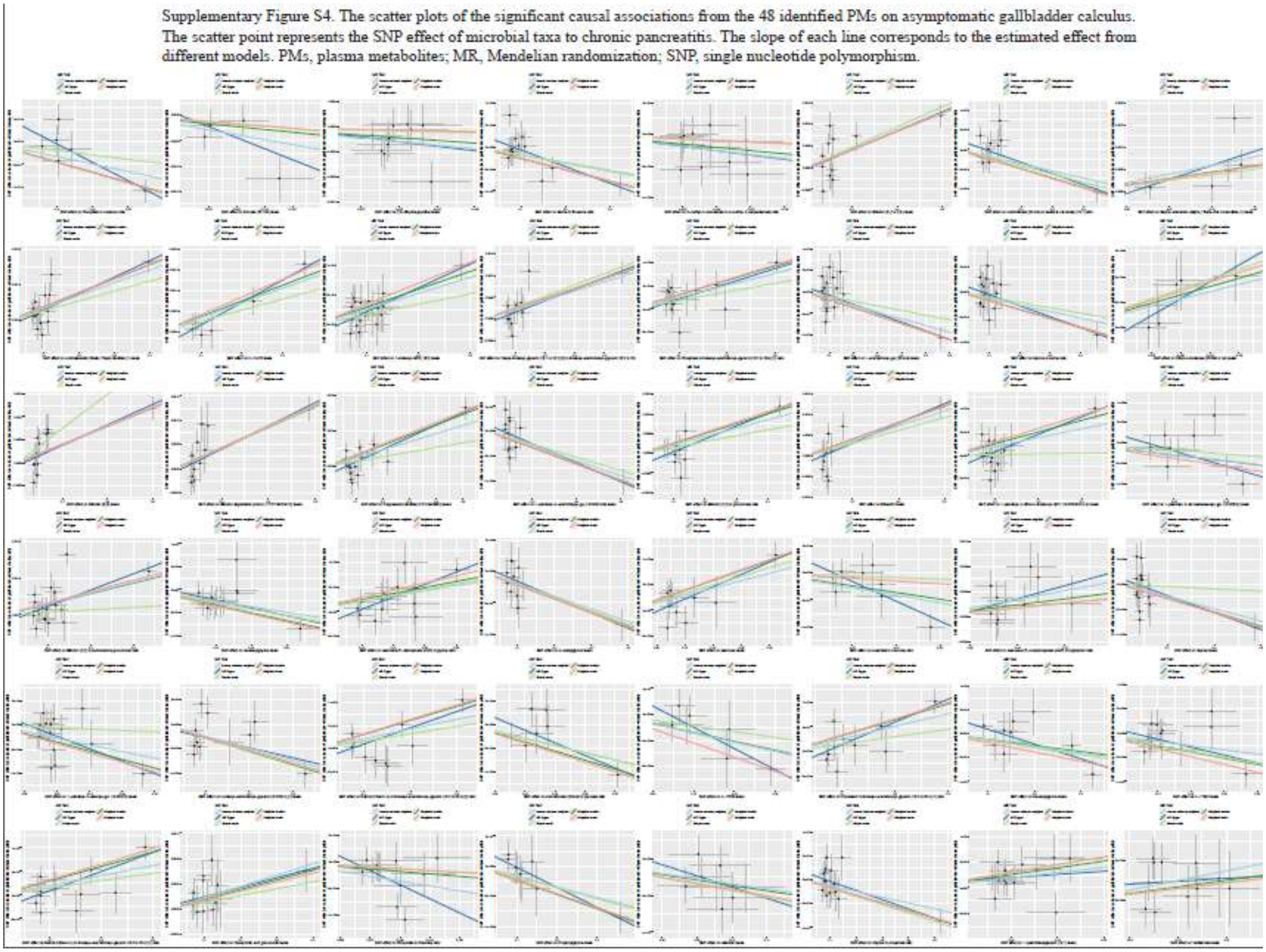

Supplementary Figure S4. The scatter plots of the significant causal associations from the 48 identified PMs on asymptomatic gallbladder calculus. The scatter point represents the SNP effect of microbial taxa to chronic pancreatitis. The slope of each line corresponds to the estimated effect from different models. PMs, plasma metabolites; MR, Mendelian randomization; SNP, single nucleotide polymorphism.

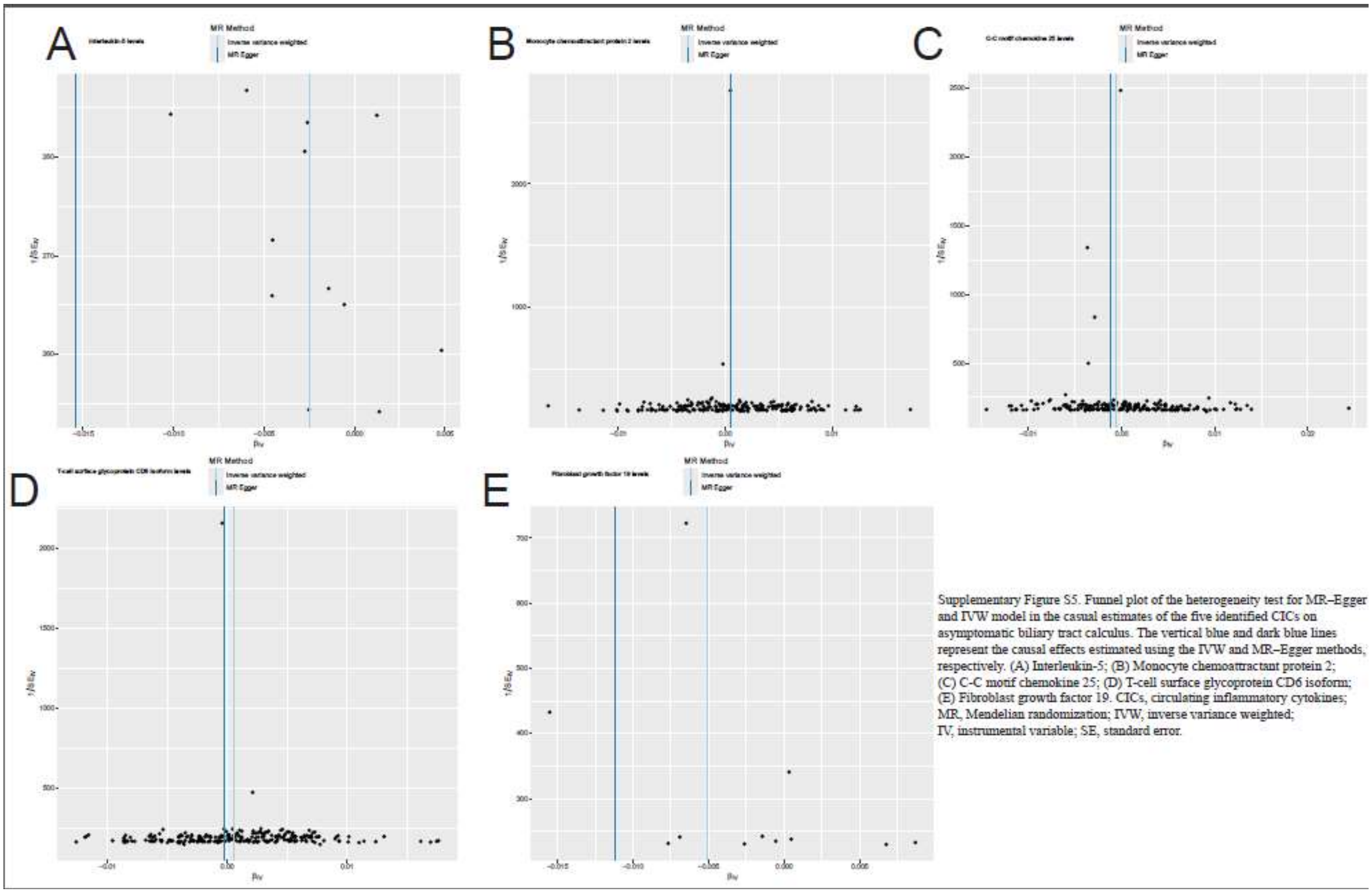

Supplementary Figure S5. Funnel plot of the heterogeneity test for MR–Egger and IVW model in the casual estimates of the five identified CICs on asymptomatic biliary tract calculus. The vertical blue and dark blue lines represent the causal effects estimated using the IVW and MR–Egger methods, respectively. (A) Interleukin-5; (B) Monocyte chemoattractant protein 2; (C) C-C motif chemokine 25; (D) T-cell surface glycoprotein CD6 isoform; (E) Fibroblast growth factor 19. CICs, circulating inflammatory cytokines; MR, Mendelian randomization; IVW, inverse variance weighted; IV, instrumental variable; SE, standard error.

Supplementary Figure S6. Funnel plot of the heterogeneity test for MR–Egger and IVW model in the casual estimates of the six identified CICs on asymptomatic gallbladder calculus. The vertical blue and dark blue lines represent the causal effects estimated using the IVW and MR–Egger methods, respectively. (A) Monocyte chemoattractant protein 2; (B) Fibroblast growth factor 19; (C) Hepatocyte growth factor; (D) TNF-beta; (E) TNF-related apoptosis-inducing ligand; (F) TNF-related activation-induced cytokine. CICs, circulating inflammatory cytokines; MR, Mendelian randomization; IVW, inverse variance weighted; IV, instrumental variable; SE, standard error.

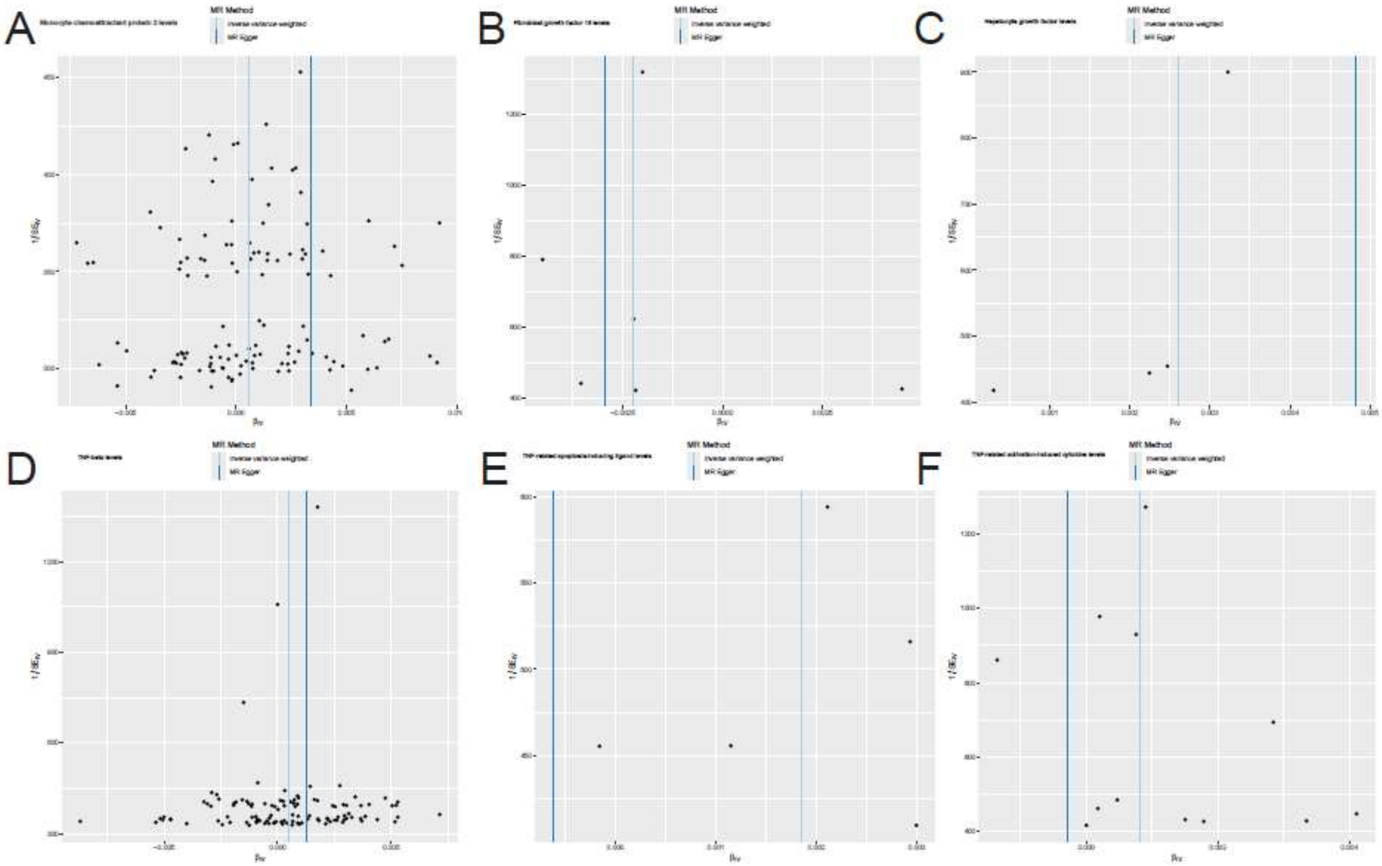

Supplementary Figure S6. Funnel plot of the heterogeneity test for MR–Egger and IVW model in the casual estimates of the six identified CICs on asymptomatic gallbladder calculus. The vertical blue and dark blue lines represent the causal effects estimated using the IVW and MR–Egger methods, respectively. (A) Monocyte chemoattractant protein 2; (B) Fibroblast growth factor 19; (C) Hepatocyte growth factor; (D) TNF-beta; (E) TNF-related apoptosis-inducing ligand; (F) TNF-related activation-induced cytokine. CICs, circulating inflammatory cytokines; MR, Mendelian randomization; IVW, inverse variance weighted; IV, instrumental variable; SE, standard error.

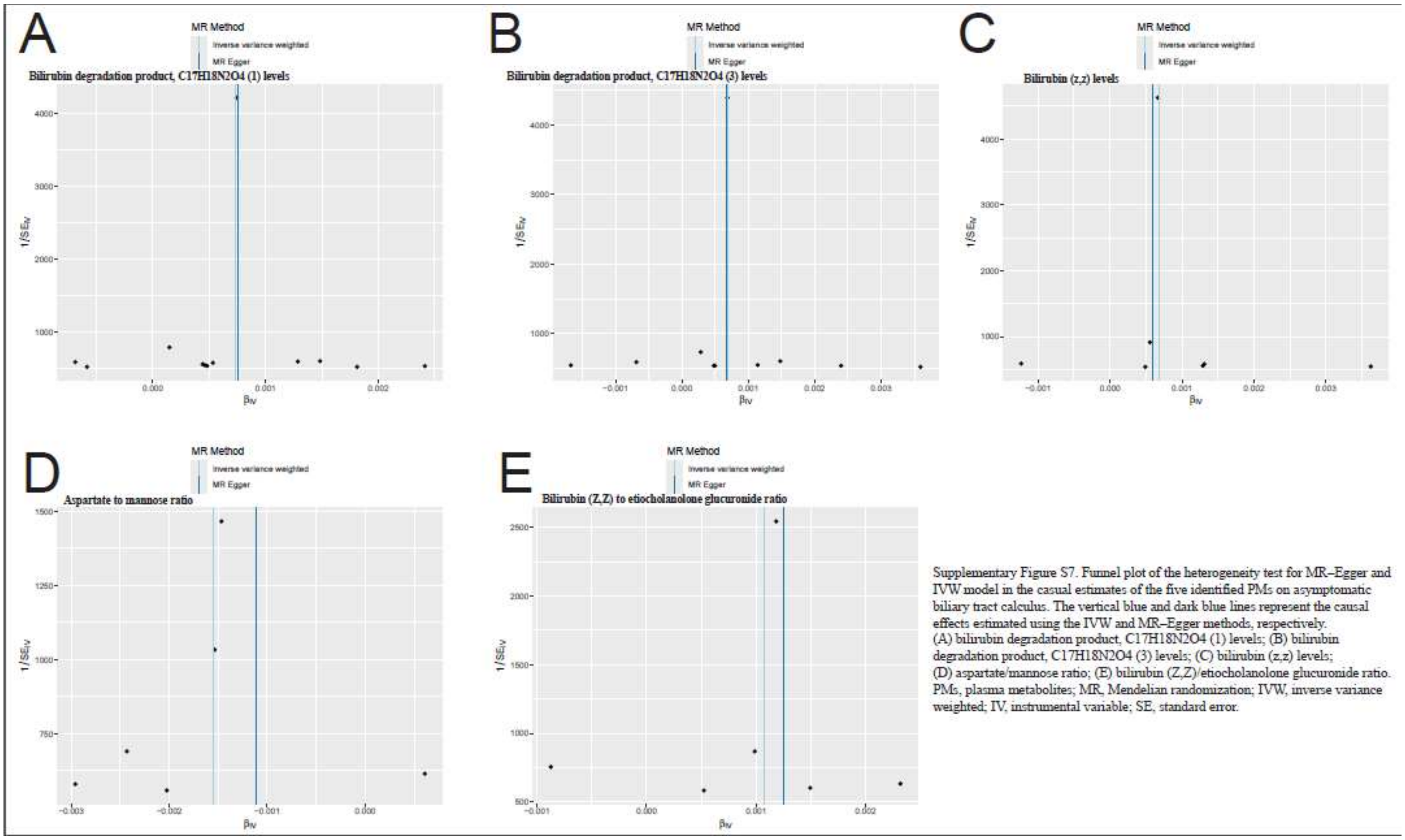

Supplementary Figure S7. Funnel plot of the heterogeneity test for MR–Egger and IVW model in the casual estimates of the five identified PMs on asymptomatic biliary tract calculus. The vertical blue and dark blue lines represent the causal effects estimated using the IVW and MR–Egger methods, respectively. (A) bilirubin degradation product, C17H18N2O4 (1) levels; (B) bilirubin degradation product, C17H18N2O4 (3) levels; (C) bilirubin (z,z) levels; (D) aspartate/mannose ratio; (E) bilirubin (Z,Z)/etiocholanolone glucuronide ratio. PMs, plasma metabolites; MR, Mendelian randomization; IVW, inverse variance weighted; IV, instrumental variable; SE, standard error.

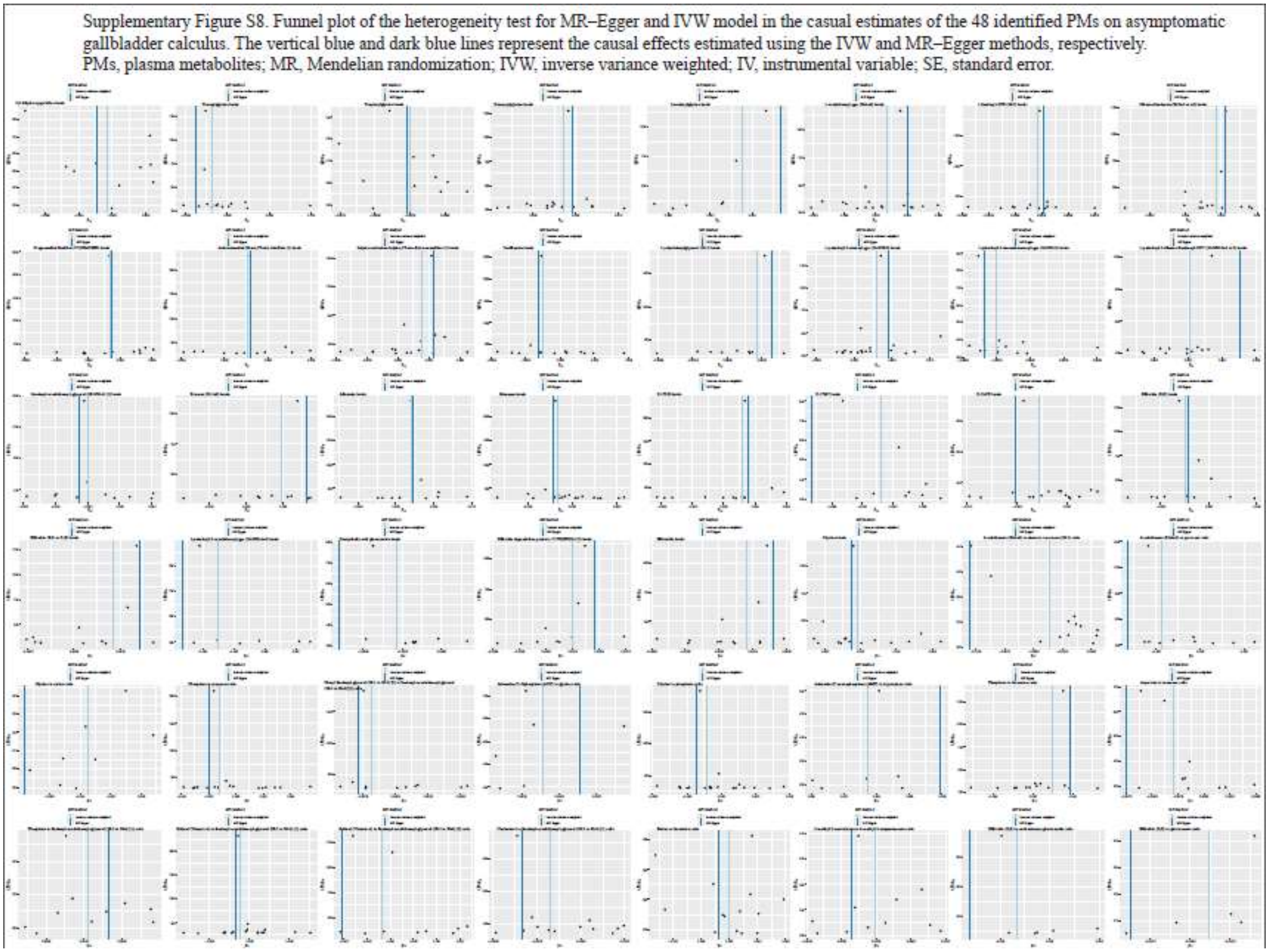

Supplementary Figure S8. Funnel plot of the heterogeneity test for MR–Egger and IVW model in the casual estimates of the 48 identified PMs on asymptomatic gallbladder calculus. The vertical blue and dark blue lines represent the causal effects estimated using the IVW and MR–Egger methods, respectively. PMs, plasma metabolites; MR, Mendelian randomization; IVW, inverse variance weighted; IV, instrumental variable; SE, standard error.

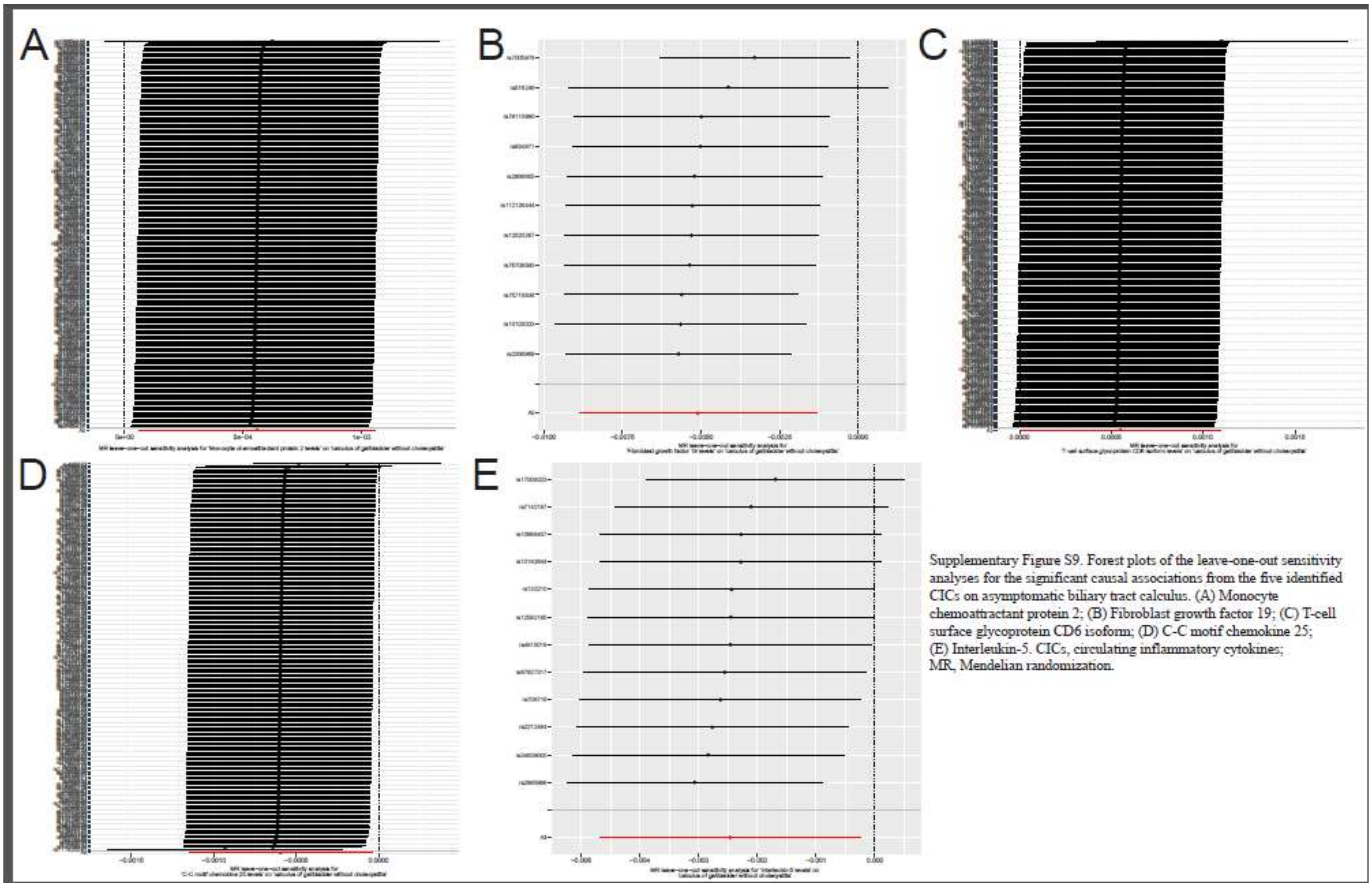

Supplementary Figure S9. Forest plots of the leave-one-out sensitivity analyses for the significant causal associations from the five identified CICs on asymptomatic biliary tract calculus. (A) Monocyte chemoattractant protein 2; (B) Fibroblast growth factor 19; (C) T-cell surface glycoprotein CD6 isoform; (D) C-C motif chemokine 25; (E) Interleukin-5. CICs, circulating inflammatory cytokines; MR, Mendelian randomization.

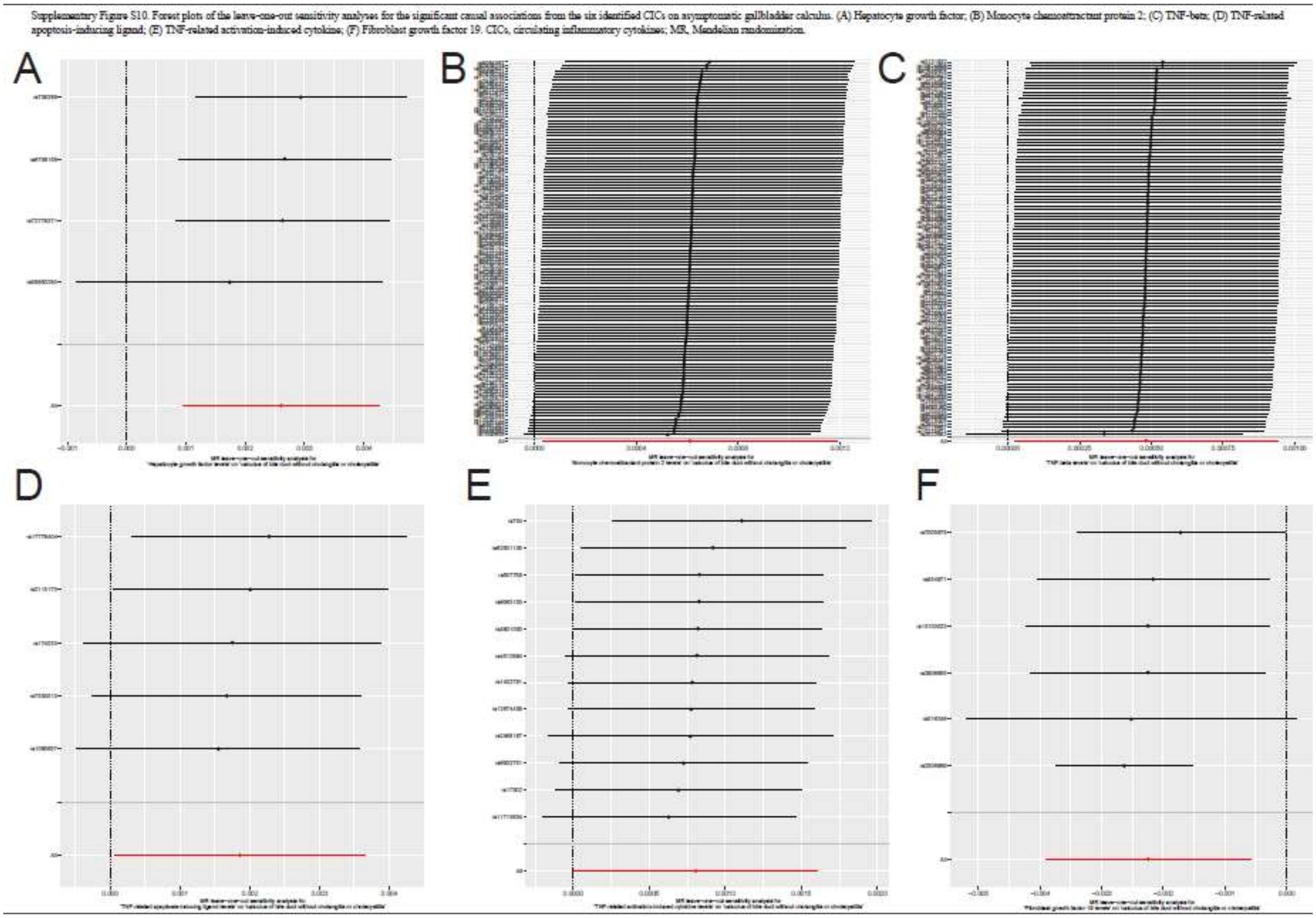

Supplementary Figure S10. Forest plots of the leave-one-out sensitivity analyses for the significant causal associations from the six identified CICs on asymptomatic gallbladder calculus. (A) Hepatocyte growth factor; (B) Monocyte chemoattractant protein 2; (C) TNF-beta; (D) TNF-related apoptosis-inducing ligand; (E) TNF-related activation-induced cytokine; (F) Fibroblast growth factor 19. CICs, circulating inflammatory cytokines; MR, Mendelian randomization.

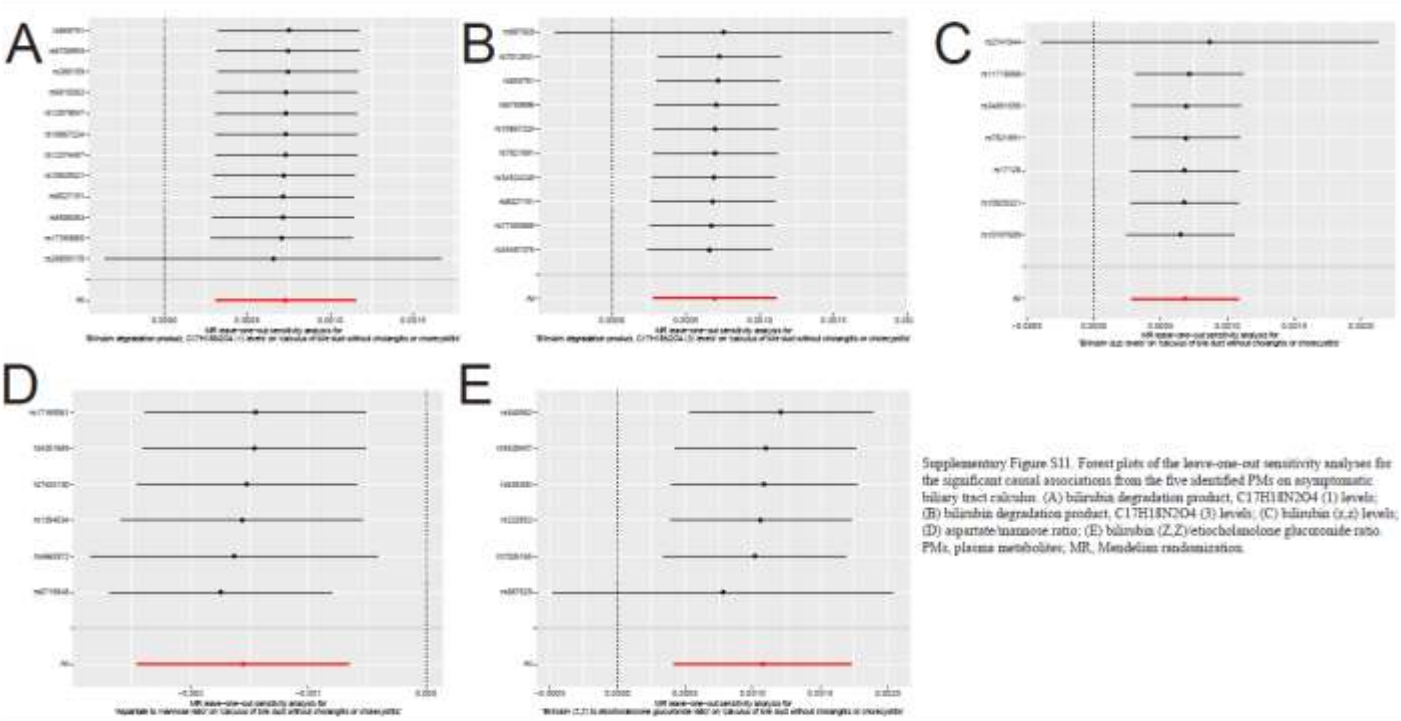

Supplementary Figure S11. Forest plots of the leave-one-out sensitivity analyses for the significant causal associations from the five identified PMs on asymptomatic biliary tract calculus. (A) bilirubin degradation product, C17H18N2O4 (1) levels; (B) bilirubin degradation product, C17H18N2O4 (3) levels; (C) bilirubin (z,z) levels; (D) aspartate/mannose ratio; (E) bilirubin (Z,Z)/etiocholanolone glucuronide ratio. PMs, plasma metabolites; MR, Mendelian randomization.

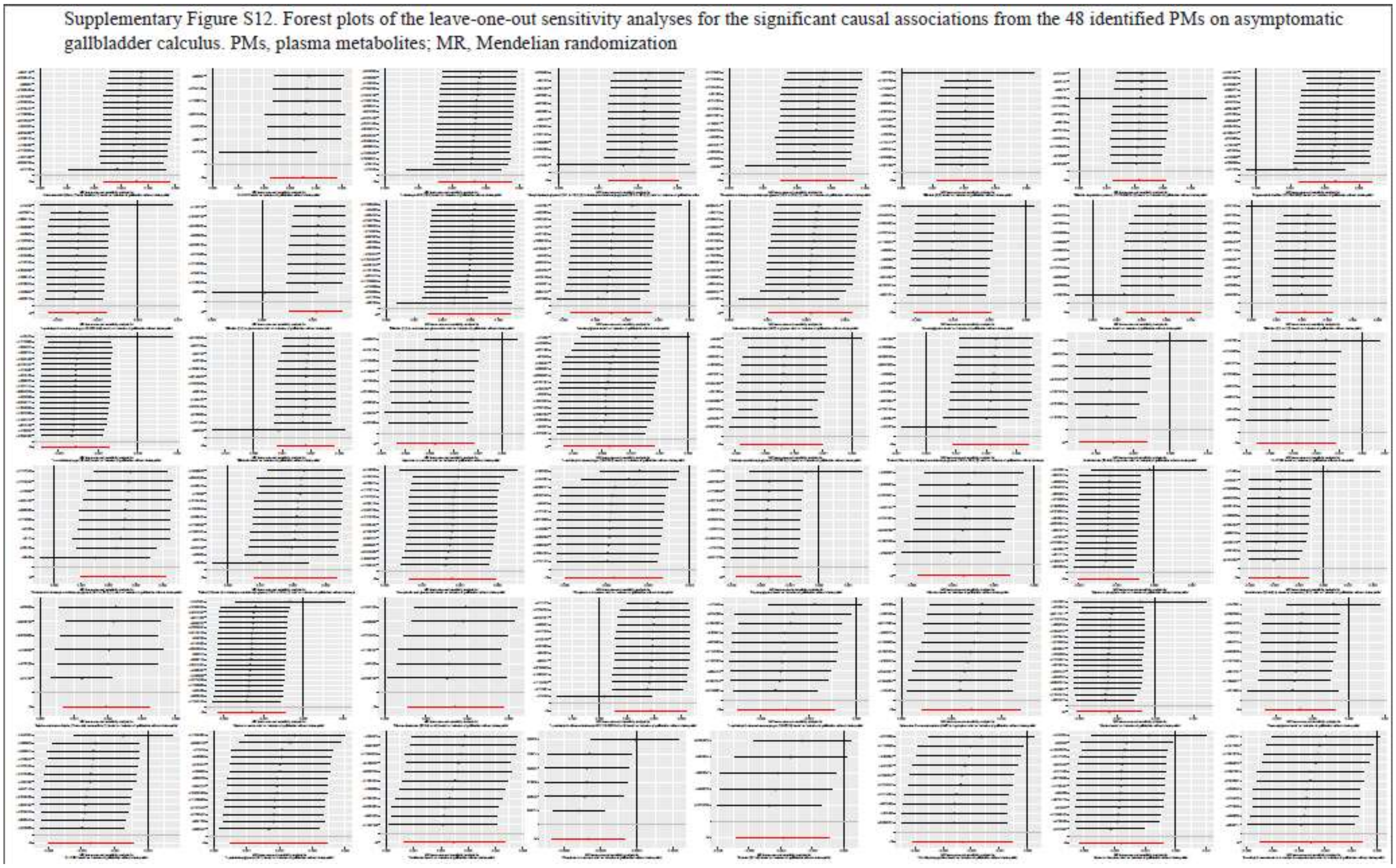

Supplementary Figure S12. Forest plots of the leave-one-out sensitivity analyses for the significant causal associations from the 48 identified PMs on asymptomatic gallbladder calculus. PMs, plasma metabolites; MR, Mendelian randomization

Supplementary Figure S12. Forest plots of the leave-one-out sensitivity analyses for the significant causal associations from the 48 identified PMs on asymptomatic gallbladder calculus. PMs, plasma metabolites; MR, Mendelian randomization.
